# Exploring novel skin biomarkers related to pruritus in dialysis patients

**DOI:** 10.1007/s11255-026-05169-2

**Published:** 2026-05-09

**Authors:** Thomas S. van Lieshout, Shannah Bennett, Jop Vreeken, Sanja Kezic, Patrick M. J. H. Kemperman, Rosalie M. Luiten, Edwin M. Spithoven, Erik L. Penne, Thomas Rustemeyer, Brigit C. van Jaarsveld, Alferso C. Abrahams

**Affiliations:** 1 Department of Nephrology, Amsterdam University Medical Center, Vrije Universiteit Amsterdam, Research Institute Amsterdam Cardiovascular Sciences, Amsterdam, The Netherlands; 2 Department of Internal Medicine, Northwest Clinics, Alkmaar, The Netherlands; 3 Department of Dermatology, Amsterdam University Medical Center, Amsterdam, The Netherlands; 4 Department of Dermatology and Netherlands Institute for Pigment Disorders, Amsterdam Institute for Infection and Immunity, Amsterdam University Medical Center, University of Amsterdam, Amsterdam, The Netherlands; 5 Department of Public and Occupational Health, Amsterdam Public Health Research Institute, Amsterdam University Medical Center, Amsterdam, The Netherlands; 6 Department of Dermatology, Dijklander Hospital, Purmerend, The Netherlands; 7 Department of Internal Medicine, Amphia Hospital, Breda, The Netherlands; 8 Department of Internal Medicine, Division of Nephrology, Rijnstate Hospital, Arnhem, The Netherlands; 9 Nephrocare Diapriva Dialysis Center, Amsterdam, The Netherlands; 10 Department of Nephrology and Hypertension, University Medical Center Utrecht, Utrecht, The Netherlands

**Keywords:** Pruritus, Dialysis, Inflammation, Biomarkers, Stratum corneum

## Abstract

**Purpose:**

Pruritus is a common and distressing symptom among dialysis patients, yet its underlying mechanisms remain incompletely understood. Most previous research has focused on systemic inflammatory markers, while little is known about local skin alterations contributing to pruritus. This study aimed to characterize skin biomarker profiles in dialysis patients with pruritus, in comparison with dialysis patients without pruritus and healthy controls, and to explore differences between pruritic and non-pruritic skin sites.

**Methods:**

In this multi-center cross-sectional exploratory study, stratum corneum samples were obtained using the tape-stripping method from 91 participants (67 dialysis patients and 24 healthy controls). Biomarkers were quantified, and adjusted linear and logistic (mixed) models were used to assess group differences and skin site variations.

**Results:**

Dialysis patients with pruritus exhibited a significantly elevated IL-1RA/IL-1*α* ratio and reduced IL-18 levels compared to non-pruritic patients. There was also a strong indication of concomitant increases in IL-1RA and IFN-*β* in pruritic skin. Across all dialysis patients, IL-8, CCL17, and CXCL10 levels were significantly lower than in healthy controls, independent of pruritus status.

**Conclusion:**

Dialysis patients with pruritus demonstrate distinct alterations in skin immune profiles suggestive of localized inflammation and potential involvement of the opioid pathway. These findings provide new insights into the inflammatory mechanisms underlying pruritus in this population and may inform the development of targeted, personalized therapies.

**Supplementary Information:**

The online version contains supplementary material available at https://doi.org/10.1007/s11255-026-05169-2.

## Introduction

‘Driving them crazy’ was the most frequent response given by dialysis patients when asked to describe their itching in a 2023 qualitative study on the burden of chronic kidney disease-associated pruritus (CKDaP) or pruritus for short [1]. The substantial burden experienced by this group of patients is consistent with other studies showing that pruritus is highly prevalent within the dialysis population, with prevalence rates around 50% [2–4]. In addition to this high prevalence, pruritus is also associated with a negative impact on health-related quality of life (HRQoL), sleep quality, and psychological symptoms [4, 5]. While awareness of this issue seems to be increasing within the medical community, the underlying pathophysiology of CKDaP is not yet clarified.

It is currently assumed that the pathophysiology of pruritus in general involves three primary mechanisms: skin alterations, neuropathy, and inflammation [6]. Among patients with chronic kidney disease (CKD), the most commonly observed skin alteration is xerosis [7–9]. Xerosis is attributed to atrophy of sebaceous glands and a reduction in the size of sweat glands, resulting in decreased hydration of the stratum corneum [10, 11]. Neuropathy, another significant contributor to pruritus, is hypothesized to result from dysregulation of the endogenous opioid system [12, 13]. Specifically, stimulation of *μ*-opioid receptors combined with suppression of *κ*-opioid receptors is believed to exacerbate pruritus. This theory is supported by evidence from studies demonstrating the efficacy of opioid receptor modulators, such as difelikefalin and naltrexone [14, 15]. Lastly, inflammation is thought to play a critical role in both the onset and persistence of pruritus. This process likely involves a complex interplay of mast cell activity (independent of histamine), T-helper cell dysregulation, and the release of inflammatory mediators [12, 16, 17]. Supporting evidence includes elevated levels of inflammatory mediators such as CRP, IL-6, and IL-31 in patients experiencing pruritus [17–19].

Previous studies indicating elevated levels of biomarkers in patients with pruritus have primarily focused on serum concentrations [17, 20, 21]. Research on skin biomarkers in pruritic skin conditions, such as psoriasis and atopic dermatitis, suggests discrepancies between levels in the skin and those in the serum [22–24]. These findings indicate that in pruritic skin conditions, inflammation may be largely confined to the skin, with certain pro-inflammatory biomarkers being primarily overexpressed locally rather than systemically. It is, therefore, essential to further explore the pathophysiology of pruritus and the role of inflammation by examining cytokine and chemokine expression within the skin itself. Gaining deeper insight into the underlying mechanisms provides an opportunity to develop new, effective therapeutic interventions that specifically target these pathways.

Therefore, we aimed to explore the role of pruritus-related skin biomarkers, previously associated with other pruritic skin conditions, in dialysis patients by investigating: (1) skin biomarker profiles in dialysis patients with and without pruritus, (2) skin biomarker profiles of the affected sites and non-affected sites within patients with pruritus, and (3) skin biomarker profiles in dialysis patients (with and without pruritus) compared with the skin biomarker profiles of healthy controls.

## Methods

### Study design and population

In this multi-center cross-sectional explorative study, dialysis patients were recruited from four different dialysis centers in the Netherlands between May and August 2023. All patients ≥18 years old with kidney failure who were on maintenance dialysis (either hemodialysis or peritoneal dialysis) were eligible for inclusion. Patients with pruritus-associated dermatological disorders, such as eczema and psoriasis, or wounds and/or skin defects other than scratch marks were excluded. Demographical data and clinical data were collected from electronic patient files. Additional data on healthy controls were obtained from a previous study on skin biomarker profiles in contact dermatitis. Participants were recruited from the Occupational Dermato-allergology Clinics of the Amsterdam UMC and had no known history of CKD or other comorbidities. Individuals were eligible for inclusion if diagnosed with occupational contact dermatitis, and samples used in this study were collected exclusively from non-lesional skin sites. All patients and controls provided written informed consent. An exemption for this study was issued by the medical research ethics committee of the Amsterdam University Medical Center in Amsterdam, The Netherlands (no.: W22_175#22.319). The study was conducted according to the principles of the Declaration of Helsinki. We reported our results in accordance with the Strengthening the Reporting of Observational Studies in Epidemiology (STROBE) guidelines (Supplementary Table S1 [25]).

### Primary and secondary outcomes

The primary outcome of this study was the concentration of several biomarkers within the skin of dialysis patients and healthy controls. These concentrations were measured using a tape-stripping technique and determined using the Meso Scale Diagnostic (MSD) technique (see below). The following biomarkers were included: IL-1*α*, IL-1RA, IL-8, IL-18, IL-21, IL-31, IFN-*β*, CCL17, and CXCL10. These biomarkers were selected based on their role within the skin and their known associations with pruritic dermatological conditions as described in Supplementary Table S2 [26–33]. Additionally, the IL-1RA/IL-1α ratio was examined as a marker of skin inflammation [34].

Secondary outcomes included the severity of pruritus and pruritus-related patient type, which were assessed using the Worst Itch Numeric Rating Scale (WI-NRS) itching scale and the Mathur Itching Questionnaire, respectively [35, 36]. The WI-NRS rates the severity of pruritus in the past 24 h on a scale from 0 to 10, 0 being “no itching” and 10 being “worst itch imaginable”. In the Mathur Itching Questionnaire, patients select one of three patient profiles that most closely resembles them. “Patient type A” reflects a patient who has no scratches, sleep problems, and agitation or sadness, “Patient type B” reflects a patient that has some, and “Patient type C” reflects a patient that often has these symptoms.

### Skin sample collection

**Fig. 1:**
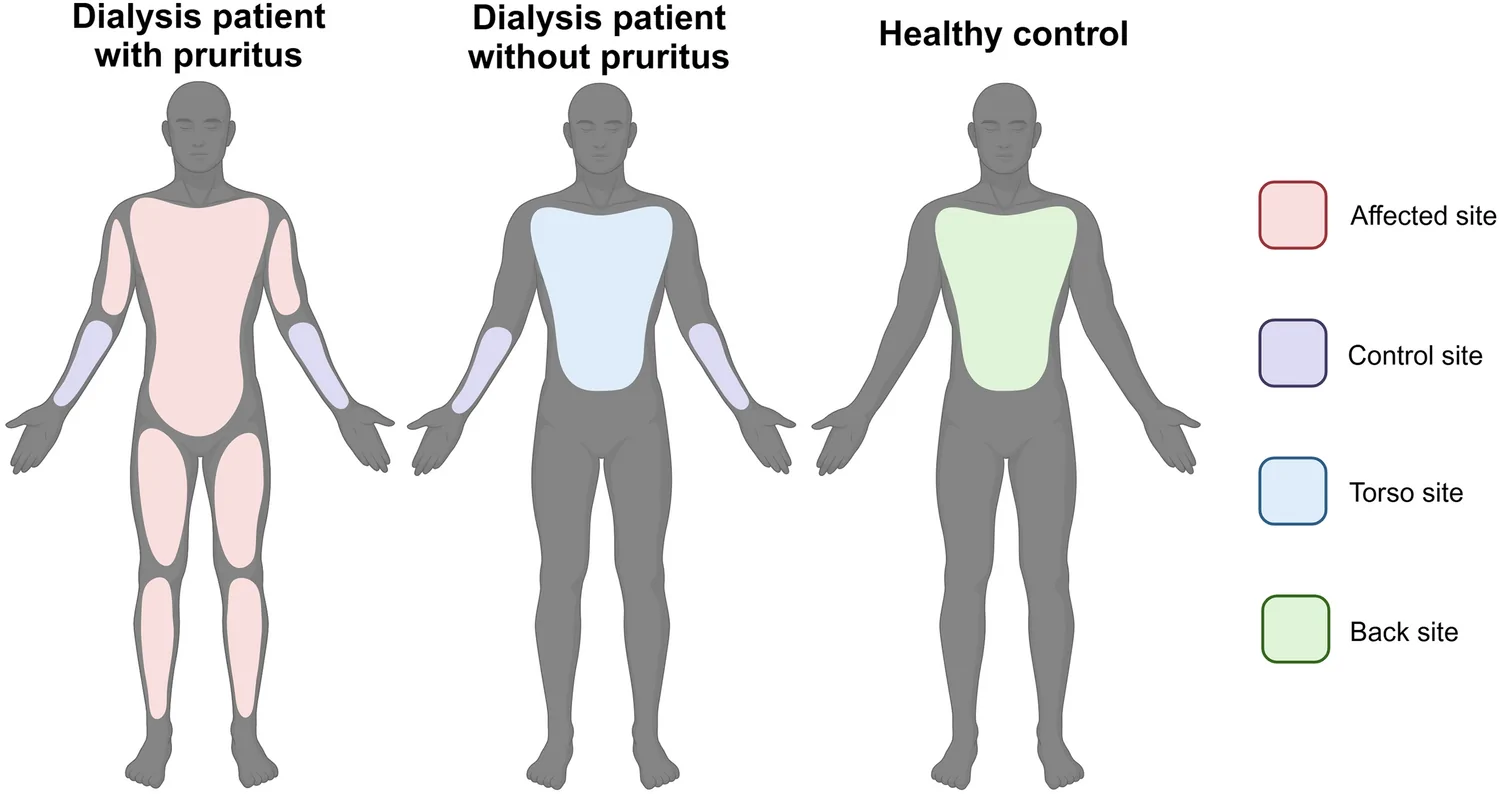
Sampling site locations for dialysis patients with and without pruritus and healthy controls. Locations in red denote the affected site in patients with pruritus, in blue denote the torso in patients without pruritus, and in green the back in healthy controls. Locations in purple denote the control (non-affected) site which was located on the forearm and was only sampled from dialysis patients

Skin samples were collected by visiting patients at their dialysis centers, and in case of peritoneal dialysis patients at the ambulant clinic or at home. Prior to collection, patients were asked if they were currently experiencing pruritus and, if so, the location of the itching. For patients reporting pruritus, samples were taken from the affected area. If pruritus was experienced at multiple areas, the site with the most severe pruritus was chosen for collection. Additionally, samples were collected from a non-affected site (i.e., control site without pruritus) on one of the forearms. For patients without pruritus, samples were obtained from the torso and the same control site on the forearm. In the healthy controls, samples taken from the back were used, with no additional control samples taken from the forearm (Fig. 1).

**Fig. 2:**
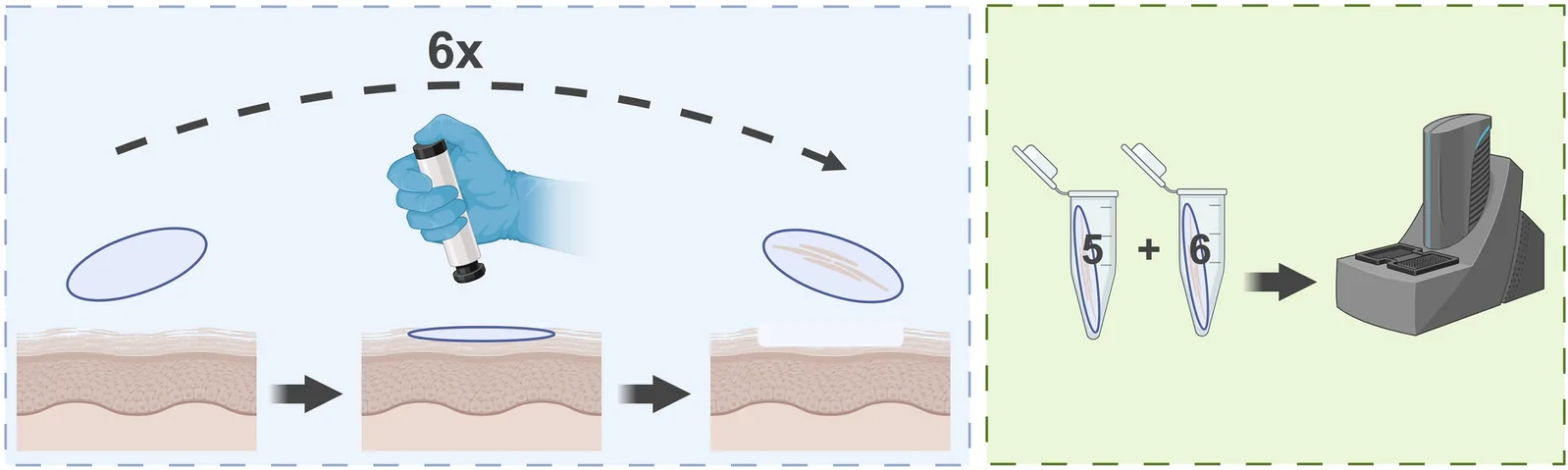
Skin sampling method. The adhesive tape strip is placed onto the surface of the skin. With the use of a pressure instrument, a standardized pressure (225 g/cm^2^) is applied to the strip. After 5 s, the strip is removed and stored in a sampling vial. By removing the strip, the outer layer of the skin (i.e., the stratum corneum) is collected as the cells within this layer adhere to the strip. This process was repeated six times ensuring to reach the deeper layer of the stratum corneum. All vials were stored at −80 °C. The fifth and sixth strips (containing the deeper layer) were used for biomarker profile analysis using the MESO QuickPlex

Skin samples were collected using adhesive tape strips, a technique that allows for the collection of the outermost layer of the skin, i.e., the stratum corneum (SC) as shown in Fig. 2. This method has been previously validated in dermatological studies [37, 38]. During the procedure, circular adhesive tape strips (3.8 cm^2^, D‐squame Discs; Monaderm, Monaco, France) were applied to the skin using a D‐Squame Pressure Instrument D500 (CuDerm Corporation, Dallas, TX, USA) for 5 s with a standardized pressure of 225 g/cm^2^. The tape strips were then carefully removed and stored in sampling vials. At each sampling site, six consecutive tape strips were collected and stored at −80 °C. The final two tape strips from each site (the fifth and sixth tape strip) were designated for biomarker profile analysis, ensuring the inclusion of the deeper layers of the stratum corneum.

### Skin biomarker analysis

The strips were extracted in 1.2 mL PBS (Life Technologies, Paisley, UK) 0.005% Tween 20 (Merck, Hohenbrunn, Germany) using an ultrasound bath (Branson 5800, Branson Ultrasonics, Ede, The Netherlands) containing ice water for 15 min at 40 kHz. Extract aliquots of 400 µl were distributed in Micronic tubes and stored at −80 °C until further analysis. Concentrations of nine cytokines and chemokines were determined using MESO QuickPlex SQ 120 (Meso Scale Diagnostics, Rockville, MA) according to the manufacturer’s instructions. To correct for differences in the amount of the SC layer between individual strips, the concentrations of the measured cytokines and chemokines were normalized to the protein concentration in each extract. The protein concentration of each extract was determined using the Pierce Micro BCA protein assay kit (Thermo Fisher Scientific, Rockford, IL, USA).

### Statistical analysis

Patient characteristics were presented for all included patients, stratified by the presence of pruritus at the time of sample collection. Continuous variables were presented as mean with standard deviation (SD), and as median with interquartile range (IQR) in the case of a skewed distribution. Categorical variables were presented as numbers with percentages.

Values below the limit of detection (LOD) were addressed using multiple imputation to minimize bias and maintain data variability [39]. Imputation was applied when <40% of the values were below the LOD. When ≥40% of the values fell below the LOD, the data were dichotomized into detectable versus non-detectable categories and analyzed accordingly [40]. Skewed data were log-transformed prior to analysis and outliers were identified and excluded using the ROUT method [41].

To analyze differences in concentrations between dialysis patients with and without pruritus and healthy controls, a linear regression model was used. For dichotomized outcomes, logistic regression models were applied. All models were adjusted for sex, age, use of immunosuppressants, and the presence of atopy. For analyzing differences between affected and non-affected sites within patients with pruritus, a linear mixed-effects model or a logistic mixed-effects model (for dichotomized outcomes) was used. These models were adjusted for the same covariates as above, with the addition of a random intercept for each participant to account for within-subject correlations. Coefficients from the linear and logistic models were back-transformed to obtain proportional differences and odds ratios, respectively. The Benjamini–Hochberg procedure was used to account for multiple testing [42]. A correlation matrix was calculated to assess intercorrelation of the included biomarkers. The results of the analyses were reported with a 95%CI or a *p* value for statistical significance.

All analyses were performed using R (version 4.2.1), SPSS version 28.0 (IBM, Armonk, NY, USA), and GraphPad Prism (version 9.5.1).

## Results

### Patient characteristics

A total of 67 patients were included in the study, of whom 34 reported pruritus at the time of sampling (Table 1) presents the characteristics of all patients, stratified by the presence of pruritus. Patients with pruritus had a lower mean age of 61.2 years (±17.3) compared to 67.8 years (±16.9) in the patients without pruritus. The group without pruritus had a longer median dialysis vintage of 33 months (IQR 19–76) compared to 26 months (IQR 16–64) in the pruritus group. Serum phosphate level were higher in the pruritus group, with a mean of 5.90 mg/dL (±2.45) compared to 4.62 mg/dL (±1.09).

**Table 1:** Dialysis patients’ characteristics

Characteristics	Total (*n* = 67)	Dialysis patients with pruritus (*n* = 34)	Dialysis patients without pruritus (*n* = 33)
Age (years), mean (SD)	64.5 (17.2)	61.2 (17.3)	67.8 (16.9)
Sex, female, *n* (%)	29 (43.3)	15 (44.1)	14 (42.4)
Body mass index (kg/m^2^)	26.6 (5.5)	25.6 (5.4)	27.7 (5.5)
Primary kidney disease, *n* (%)			
Glomerular disease	14 (21.2)	9 (26.5)	5 (15.6)
Tubulointerstitial disease	8 (12.1)	4 (11.8)	4 (12.5)
Diabetes mellitus	15 (22.7)	6 (17.6)	9 (28.1)
Hypertension/renal vascular disease	21 (31.8)	13 (38.2)	8 (25.0)
Other systemic disease affecting the kidney	0 (0.0)	0 (0.0)	0 (0.0)
Familial/hereditary nephropathies	4 (6.1)	1 (2.9)	3 (9.4)
Miscellaneous renal disorders	4 (6.1)	1 (2.9)	3 (9.4)
Charlson comorbidity index	4 [3, 6]	4 [3, 6]	4 [3, 6]
Pruritus-associated comorbidity, *n* (%)			
Liver disease	0 (0.0)	0 (0.0)	0 (0.0)
Previous malignancy	15 (22.4)	9 (26.5)	6 (18.2)
Systemic disease	5 (7.5)	1 (2.9)	4 (12.1)
Atopy, *n* (%)			
Asthma	4 (6.0)	2 (5.9)	2 (6.1)
Atopic eczema (not active)	6 (9.0)	3 (8.8)	3 (9.1)
Allergic rhinitis	10 (14.9)	5 (14.7)	5 (15.2)
Food allergy	3 (4.5)	2 (5.9)	1 (3.0)
Anti-pruritic medication, *n* (%)			
Sedating anti-histamine	4 (6.0)	2 (5.9)	2 (6.1)
Non-sedating antihistamine	4 (6.0)	1 (2.9)	3 (9.1)
Topical steroids	7 (10.4)	5 (14.7)	2 (6.1)
Systemic steroids	8 (11.9)	1 (2.9)	7 (21.2)
Gabapentinoids	6 (9.0)	2 (5.9)	4 (12.1)
Emollients	17 (25.4)	11 (32.4)	6 (18.2)
Opioid modulators	0 (0.0)	0 (0.0)	0 (0.0)
UVB phototherapy	1 (1.5)	1 (2.9)	0 (0.0)
TCAs	4 (6.0)	3 (8.8)	1 (3.0)
SSRIs	1 (1.5)	1 (2.9)	1 (3.0)
None	30 (44.8)	17 (50.0)	13 (39.4)
Dialysis modality, *n* (%)			
Hemodialysis	54 (80.6)	27 (79.4)	27 (81.8)
Peritoneal dialysis	13 (19.4)	7 (20.6)	6 (18.2)
Dialysis vintage (months)	31 [18, 72]	26 [16, 64]	33 [19,76]
Dialysis adequacy (stdKtV), median [IQR]^a^	2.37 [2.08, 2.95]	2.50 [2.09, 3.14]	2.32 [2.06, 2.87]
Residual diuresis (>100 ml/day), *n* (%)	33 (49.3)	17 (50.0)	16 (48.5)
Prior kidney transplant, *n* (%)	12 (17.9)	5 (14.7)	7 (21.2)
Immunosuppressant use, *n* (%)^b^	16 (23.9)	7 (20.6)	9 (27.3)
Serum CRP level (mg/L), median [IQR]	3.6 [1.5, 10.0]	3.8 [2.0,10.0]	3.5 [1.2, 11.3]
Serum calcium level (mg/dL), mean (SD)	9.36 (0.64)	9.44 (0.76)	9.32 (0.52)
Serum magnesium level (mg/dL), mean (SD)	2.04 (0.40)	2.02 (0.36)	2.10 (0.56)
Serum phosphate level (mg/dL), mean (SD)	5.27 (1.98)	5.90 (2.45)	4.62 (1.09)
Serum PTH level (pg/mL), median [IQR]	306.8 [210.9, 512.8]	326.4 [196.7, 562.5]	302.7 [213.0, 469.8]

Missing values: primary kidney disease, *n* = 1 (1.5%); dialysis adequacy, *n* = 7 (10.4%); serum CRP level, *n* = 17 (25.4%); serum calcium level, *n* = 9 (13.4%), serum magnesium level, *n* = 42 (62.7%); serum phosphate level, *n* = 1 (1.5%); serum PTH level, *n* = 1 (1.5%).

^a^KT/V is a measure of dialysis adequacy, K = dialyzer urea clearance, T = dialysis time, and V = patient’s total body water volume. For hemodialysis patients, standardized KT/V (dialysis dose adjusted for frequency) was used; for peritoneal dialysis patients, weekly KT/V was used.

^b^Immunosuppressant used was defined as a medication that reduces activity of the immune system, including both systemic and topical medications.

*SD* standard deviation, *IQR* interquartile range, *UVB* ultraviolet B, *TCA* tricyclic antidepressant, *SSRI* selective serotonin reuptake inhibitor, *CRP* C-reactive protein, *PTH* parathyroid hormone.

### Pruritus-related outcomes

**Fig. 3:**
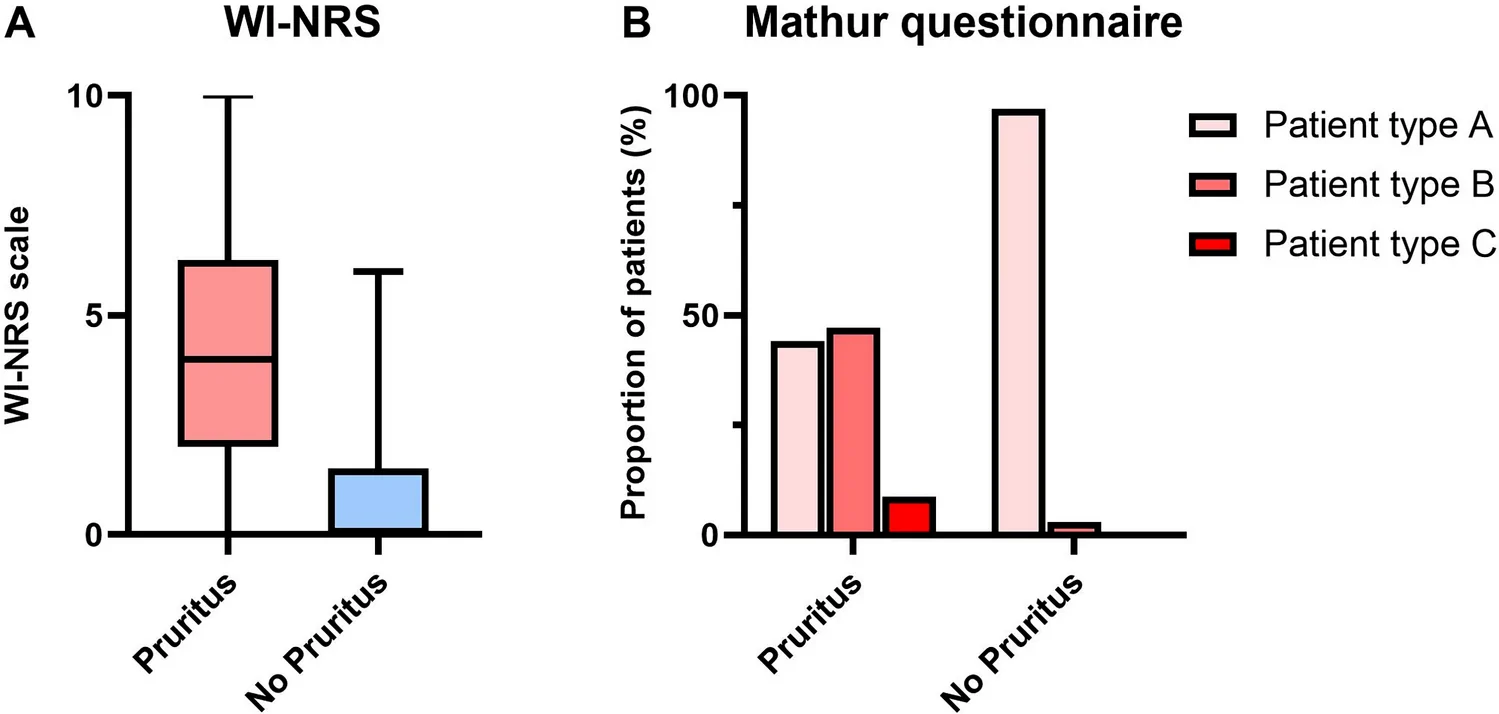
Pruritus severity and pruritus-related patient profile in dialysis patients with and without pruritus. (**A**) The severity of pruritus measured by the Worst Itch Numeric Rating Scale (WI-NRS) on a scale from 0 to 10 in dialysis patients with and without pruritus. (**B**) The pruritus-related patient profiles in dialysis patients with and without pruritus. ‘Patient type A’ reflects a patient who has no scratches, sleep problems, and agitation or sadness, ‘Patient type B’ reflects a patient who has some scratches, some sleep problems, and some agitation or sadness, and ‘Patient type C’ reflects a patient who often has scratches, sleep problems, and agitation or sadness

Patients with pruritus most commonly reported the torso as the primary affected site (67.6%), followed by the lower extremities (26.5%) and the upper extremities (5.9%). Among these patients, dry skin was observed in 52.9%, scratches were present in 8.8%, and no skin abnormalities in the remaining patients. The median severity of pruritus measured with the WI-NRS questionnaire was scored at 4.0 (IQR 2.0–6.3) for patients with pruritus, compared to 0.0 (IQR 0.0–1.5) for those without pruritus (Fig. 3). Within the group of patients with pruritus, 19 patients (55.9%) had a score of 3 to 7 (moderate pruritus) and 6 patients (17.6%) had a score of 8 or higher (severe pruritus). According to the Mathur questionnaire, patients with pruritus most often identified as type B (some scratches, some sleep problems and some agitation or sadness).

### Differences in skin biomarker profiles between dialysis patients with and without pruritus

A visual overview of the median concentrations of skin biomarkers (including IQR and outliers) is presented in supplemental Fig. S1 (for exact values on the descriptives and analyses see Tables S3 and S4). IL-21 and IL-31 were below LOD in 100.0% and 95.5% of all samples, respectively, and were consequently excluded from further analyses. IFN-*β* had 44.3% below LOD and CXCL10 had 62.0% below LOD, and were dichotomized in detectable and non-detectable and analyzed accordingly (Table S5 in the supplementary material).

**Fig. 4:**
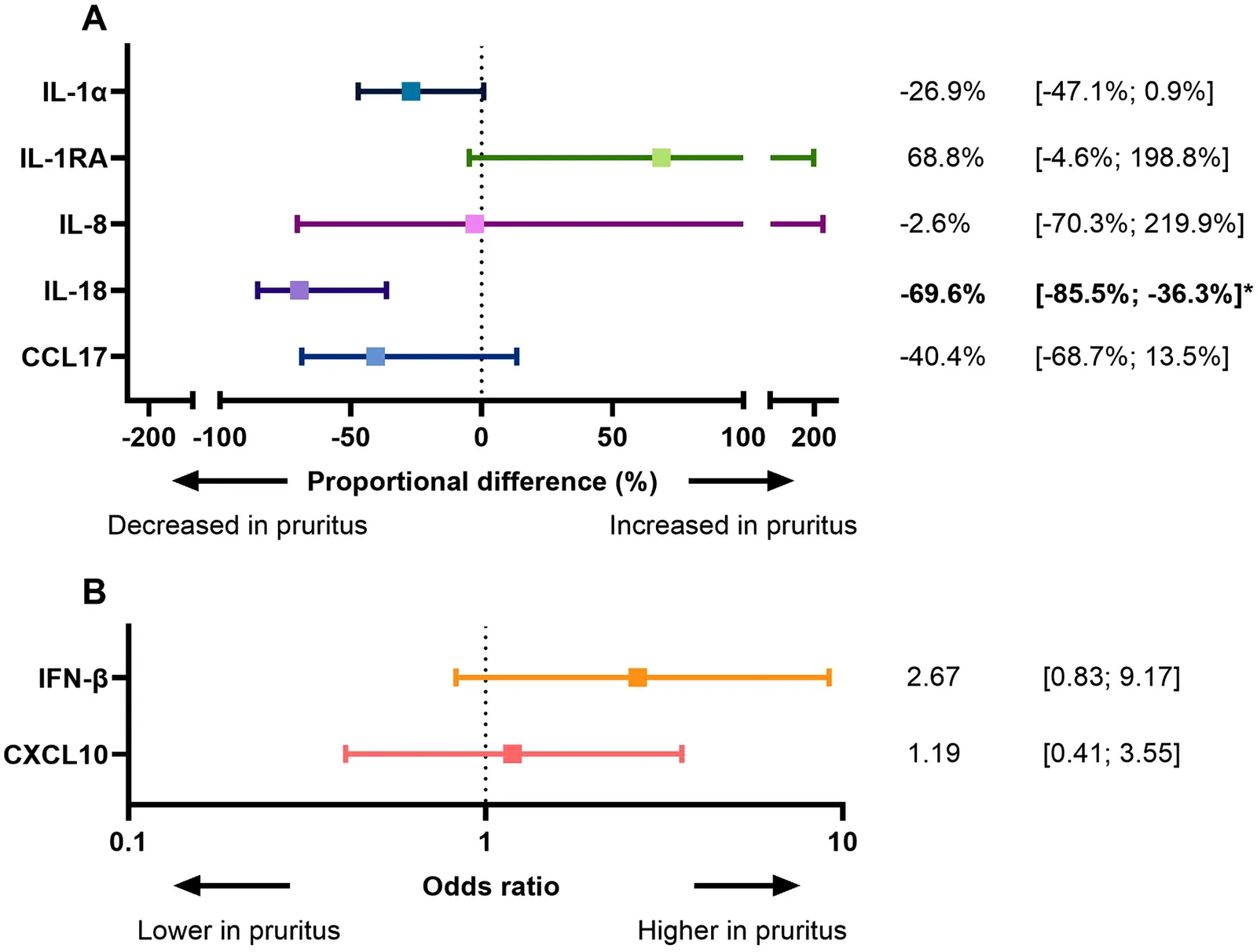
Proportional differences and odds ratios in skin biomarkers between dialysis patients with and without pruritus. (**A**) Proportional difference in skin biomarker concentration of patients with pruritus compared to patients without pruritus. Differences were calculated using adjusted linear regression models. Models were adjusted for sex, age, use of immunosuppressants, and the presence of atopy. The dotted line represents a proportional difference of 0 compared to patients without pruritus. Proportional differences were presented in percentages with a 95% confidence interval. (**B**) Odds ratios on detectability of skin biomarker concentrations of patients with pruritus compared to patients without pruritus. Odds ratios were calculated using adjusted logistic regression models. The dotted line represents an odds ratio of 1 between patients with and without pruritus. The odds ratios were presented with a 95% confidence interval. *Significant difference (*p* < 0.05) after adjustment for multiple testing. *IL-1α* interleukin 1-alpha, *IL-1RA* interleukin-1 receptor antagonist, *IL-8* interleukin 8, *IL-18* interleukin 18, *IFN-β* interferon beta, *CCL17* CC motif chemokine ligand 17, *CXCL10* CXC motif chemokine ligand 10

**Fig. 5:**
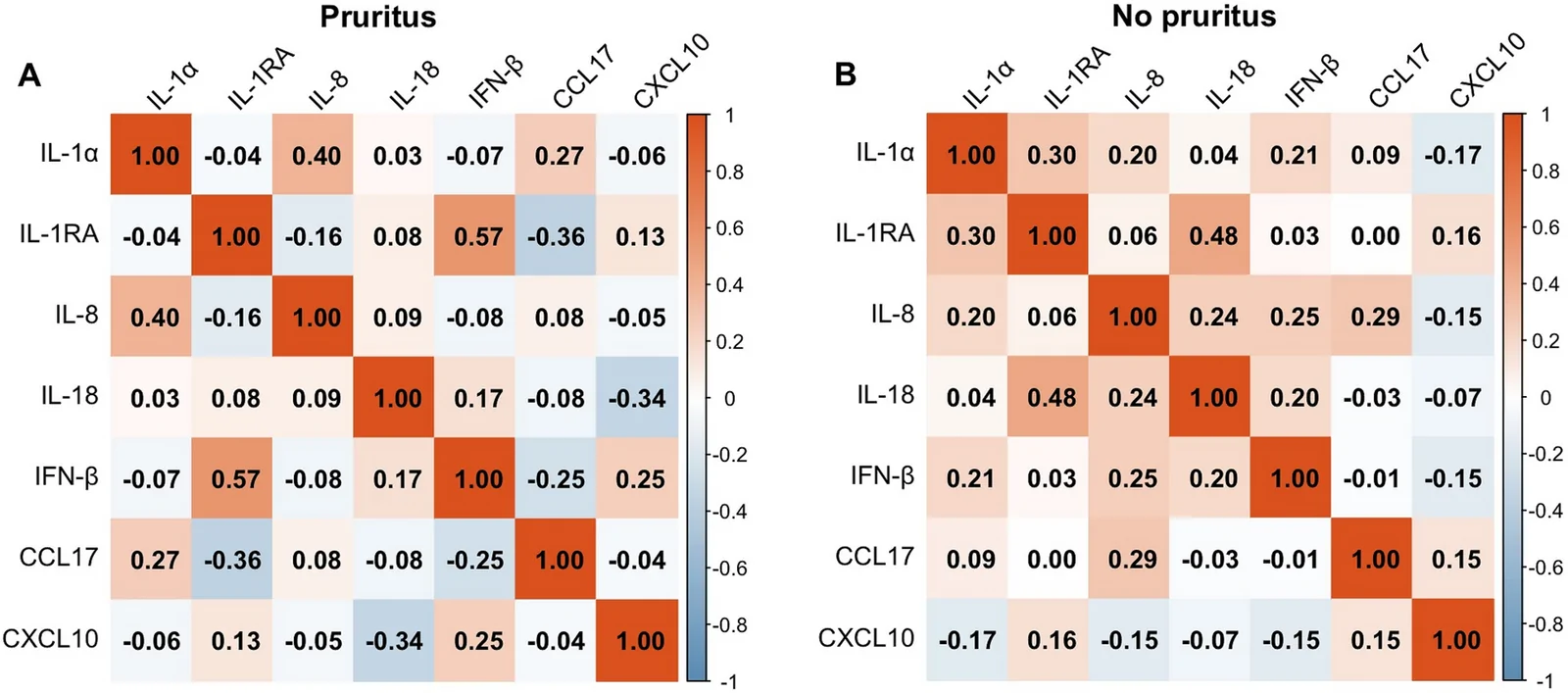
Correlation between skin biomarkers in dialysis patients with and without pruritus. (**A**) Correlation plot of the skin biomarkers in patients with pruritus. (**B**) Correlation plot of the skin biomarkers in patients without pruritus. Positive correlations are reflected by red, negative correlations by blue, and no correlations by white. A correlation of 1 represents a maximum positive correlation between biomarkers, and a correlation of −1 represents a maximum negative correlation. *IL-1α* interleukin 1-alpha, *IL-1RA* interleukin-1 receptor antagonist, *IL-8* interleukin 8, *IL-18* interleukin 18, *IFN-β* interferon beta, *CCL17* CC motif chemokine ligand 17, *CXCL10* CXC motif chemokine ligand 10

**Fig. 6:**
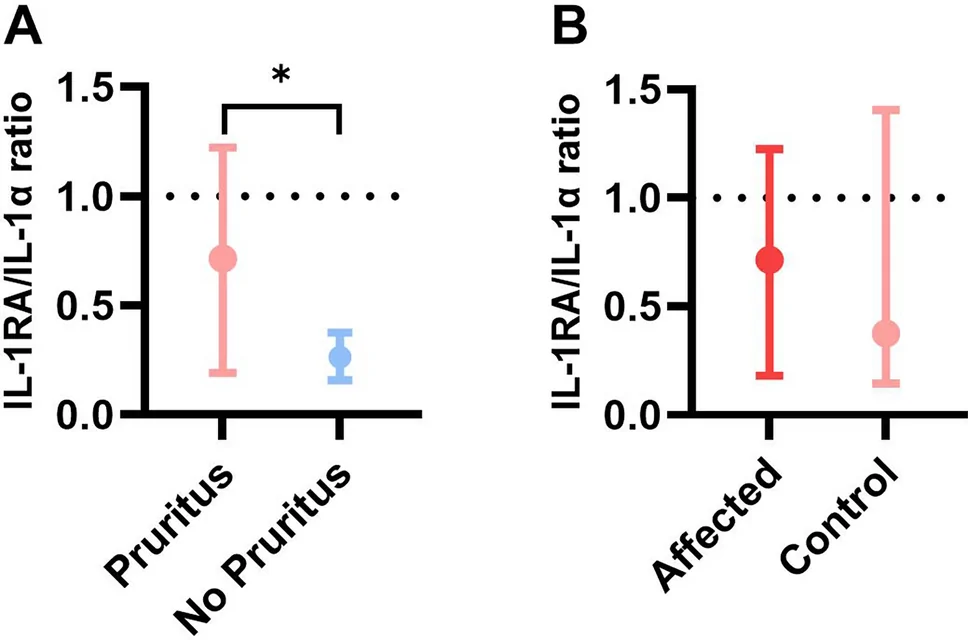
Differences in IL-1RA/IL-1*α* ratio between dialysis patients with and without pruritus and between the affected and control sites in dialysis patients with pruritus. (**A**) Difference in IL-1RA/IL-1*α* ratio between patients with and without pruritus. The ratios were analyzed using an adjusted linear regression model. Models were adjusted for sex, age, use of immunosuppressants, and the presence of atopy. (**B**) Difference in IL-1RA/IL-1*α* ratio between the affected and control (non-affected) sites in patients with pruritus. The ratios were analyzed using an adjusted linear mixed model. Models were adjusted for sex, age, use of immunosuppressants, and the presence of atopy, with the addition of a random intercept for participants. The dotted line represents a ratio of 1, corresponding to equal concentrations of IL-1RA and IL-1α. Statistical significance: ∗, *p* < 0.05. *IL-1α* interleukin 1-alpha, *IL-1RA* interleukin-1 receptor antagonist

IL-18 levels were significantly lower in patients with pruritus compared to those without, with a median of 0.08 pg/µg protein (IQR 0.03–0.32) compared to 0.29 pg/µg protein (IQR 0.08–0.58), respectively. While other biomarkers did not exhibit statistically significant differences, Fig. 4 shows that IL-1RA concentrations were 68.8% higher in patients with pruritus. The odds of having detectable levels of IFN-*β* were 2.67 (95%CI 0.83; 9.17) times higher in patients with pruritus. In Fig. 5, a positive correlation coefficient of 0.57 can be seen between IL-1RA and IFN-*β* in patients with pruritus, compared to no correlation with a coefficient of 0.03 in those without. A significant difference was observed in the IL-1RA/IL-1*α* ratio, with a ratio of 0.71 (95%CI 0.19; 1.22) in patients with pruritus versus 0.26 (95%CI 0.16; 0.38) in those without (Fig. 6).

### Differences in skin biomarker profiles between the affected and non-affected sites in dialysis patients with pruritus

**Fig. 7:**
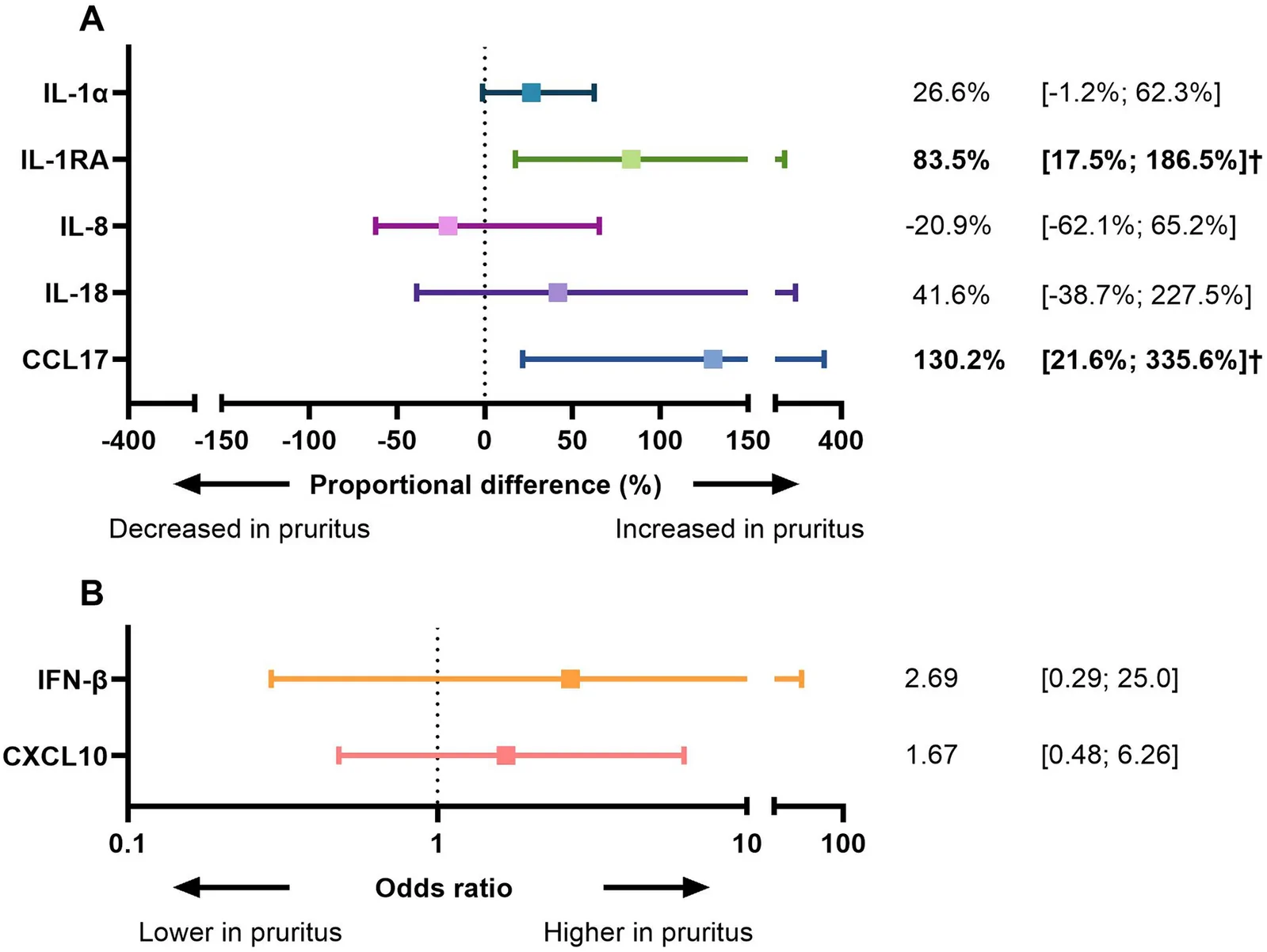
Proportional differences and odds ratios in skin biomarkers between the affected and non-affected sites in dialysis patients with pruritus. (**A**) Proportional difference in skin biomarker concentration of the affected site compared to the non-affected site within patients with pruritus. Differences were calculated using adjusted linear mixed models. The dotted line represents a proportional difference of 0 compared to the non-affected site. Proportional differences were presented in percentages with a 95% confidence interval. (**B**) Odds ratios on detectability of skin biomarker concentrations of the affected site compared to the non-affected site in patients with pruritus. Odds ratios were calculated using adjusted logistic mixed models. The dotted line represents an odds ratio of 1 between the affected and non-affected sites. The odds ratios were presented with a 95% confidence interval. †Significant difference (*p* < 0.05) disappeared after correcting for multiple testing. IL-1*α* interleukin 1-alpha, *IL-1RA* interleukin-1 receptor antagonist, *IL-8* interleukin 8, *IL-18* interleukin 18, *IFN-β* interferon beta, *CCL17* CC motif chemokine ligand 17, *CXCL10* CXC motif chemokine ligand 10

Supplemental Figure S2 shows a visual overview of the median concentration of skin biomarkers (including IQR and outliers) in the affected skin of patients with pruritus and in the skin of the non-affected site (Tables S6 and S7). CCL17 and IL-1RA concentrations were significantly higher in the affected site compared to the control site (Fig. 7). These significant differences disappeared after adjustment for multiple testing, with adjusted *p* values of 0.062 for both. In addition, in the patients without pruritus, CCL17 concentrations were also higher in samples taken from the torso compared to the control site on the forearm (Table S6). IFN-*β* showed a non-significant odds ratio of 2.69 (95%CI 0.29; 25.01) of being detectable in the affected site compared to the non-affected site. Other biomarkers showed no statistical significant difference.

### Skin biomarker profiles of dialysis patients with and without pruritus and of healthy controls

A total of 24 healthy controls were included in the study, of whom 18 were women (75.0%), with a mean age of 44.1 years (±17.1). Among these controls, the following available biomarkers were assessed: IL-1*α*, IL-1RA, IL-8, CCL17, and CXCL10 (Fig. S1 and Table S4). Notably, IL-1*α* levels were significantly higher in dialysis patients without pruritus compared to healthy controls, with a median value of 120.40 pg/µg protein (IQR 76.79–163.15) versus 66.47 (IQR 33.96–86.78). Furthermore, both IL-8 and CCL17 levels were significantly lower in the skin dialysis patients compared to healthy controls, regardless of the presence of pruritus. Specifically, the median IL-8 concentration was 0.050 pg/µg protein (IQR 0.018–0.111) in healthy controls compared to 0.008 (IQR 0.002–0.013) in dialysis patients with pruritus and 0.005 (IQR 0.001–0.034) in those without. Similarly, CCL17 levels were 0.038 pg/µg protein (IQR 0.028–0.091) in healthy controls versus 0.002 (IQR 0.001–0.003) in dialysis patients with pruritus and 0.003 (IQR 0.002–0.004) in those without. CXCL10 was significantly less detectable in dialysis patients with and without pruritus compared to healthy controls, with odds ratios of 0.027 (95%CI 0.001; 0.497) and 0.023 (95%CI 0.001; 0.443), respectively.

## Discussion

This is the first study to investigate the presence of inflammation-related biomarkers in the stratum corneum of patients with CKDaP. Within our included population, the pruritus was predominantly localized on the torso, and in half of the cases, it was accompanied by dry skin. Our results showed differences in skin biomarkers between patients with and without pruritus, as well as between affected and non-affected sites in patients with pruritus. First, we observed a significantly increased IL-1RA/IL-1*α* ratio and significantly lower levels of IL-18 in patients with pruritus compared to those without. Additionally, we identified a strong indication of a concurrent increase in IL-1RA and IFN-*β* levels, based on an increase in patients with pruritus and a local increase in the affected site. Furthermore, a higher intercorrelation between these two biomarkers was observed in patients with pruritus. Based on the findings of this study, a shared underlying mechanism in the regulation of IL-1 cytokine members such as IL-1*α* and IL-18 in the development of pruritus in these patients is plausible. Lastly, independent of the presence of pruritus, we found significantly lower levels of IL-8, CCL17, and CXCL10 in the skin of dialysis patients compared to healthy controls, suggesting an impact of kidney failure itself or dialysis on these biomarkers within the skin.

To investigate the role of skin inflammation in pruritus, we began by examining biomarkers known to be associated with disease activity in pruritic skin conditions such as psoriasis and atopic dermatitis (Table 1). These dermatological conditions share common features, including inflammation driven by autoimmune or dysregulated immune responses and impaired skin barrier function [43, 44]. It is well-known that CKD, and particularly kidney failure, impacts the immune system, leading to both immunosuppression and immune activation [45]. This immune dysregulation could hypothetically result in similar pathophysiological changes in the skin as observed in pruritic skin diseases.

Based on our findings, we identified certain similarities with these skin conditions, but also key differences. For example, atopic dermatitis is often characterized by upregulation of CCL17, a type-2 inflammatory mediator [46]. However, in our study, CCL17 was not differentially expressed between dialysis patients with and without pruritus. Elevated levels of IL-8 and IL-18 are typically observed in active psoriasis and atopic dermatitis; however, we found no difference in IL-8 levels, and IL-18 levels were even lower in patients with pruritus [47–49]. In contrast, we did observe a significantly increased IL-1RA/IL-1*α* ratio in patients with pruritus, a ratio that has been shown to reflect increased skin inflammation in several skin conditions [34]. Our findings suggest that while there is clear evidence of increased inflammation in the skin of patients with pruritus, the underlying pathophysiological pathways driving pruritus in this population appear to differ from those of other known pruritic skin conditions.

Based on our findings, we strongly suspect that IL-1RA and IFN-*β* jointly contribute to the development of pruritus in dialysis patients. As previously mentioned, we observed elevated levels of IL-1RA and IFN-*β* in the skin of patients with pruritus compared to those without, as well as in affected versus non-affected skin sites. Although these differences were not statistically significant, the increased intercorrelation between these two biomarkers in patients with pruritus compared to those without suggests that the lack of statistical significance may stem from insufficient power.

IL-1RA is a cytokine primarily produced by keratinocytes and exhibits anti-inflammatory properties in the skin [26, 34]. As discussed, elevated IL-1RA levels and an imbalance with IL-1*α*, resulting in an increased IL-1RA/IL-1*α* ratio, are commonly observed in inflammatory skin conditions. When these skin conditions are effectively treated, the IL-1RA/IL-1*α* ratio decreases, thus making it a dynamic biomarker reflecting local inflammation in the skin [34], Similarly, IFN-*β*, a type I interferon also produced by keratinocytes, is frequently implicated in diseases such as psoriasis and in wound healing processes [50]. Furthermore, IFN-*β* treatment in patients with multiple sclerosis is associated with adverse cutaneous effects [51]. Interestingly, increased levels of both IL-1RA and IFN-*β* have been strongly associated with the activation of *μ*-opioid receptors in studies exploring the role of inflammatory pain development [52, 53]. As previously discussed, activation of *μ*-opioid receptors coupled with suppression of *κ*-opioid receptors seems to play an important role in the neuropathway of pruritus [13, 14, 54]. These findings suggest that inflammatory and neuropathic pathways may not be distinct mechanisms driving pruritus. Instead, inflammation in the skin may trigger *μ*-opioid receptor activation, thereby initiating the neuropathic pathway. To substantiate this hypothesis, future research should investigate whether the presence of these biomarkers in dialysis patients with pruritus is directly associated with *μ*-opioid receptor activation. If confirmed, this could provide new therapeutic targets for the management of pruritus.

IL-31 is often referred to as the ‘itchy’ cytokine due to its association with a variety of pruritus-related conditions, including both dermatological diseases and allergic reactions. The prevailing theory suggests that IL-31, released by T helper 2 cells, triggers neuroimmune stimulation that results in the sensation of itch [55]. Elevated serum levels of IL-31 are also frequently reported in patients with CKDaP [19]. In our study, however, IL-31 was virtually undetectable in the skin. This finding aligns with other studies on pruritic skin diseases, which also report highly variable and often low concentrations of IL-31 in the skin, while systemic levels are typically elevated [56]. These results suggest that IL-31 may primarily play a role in pruritus through systemic circulation or possibly in the deeper layers of the skin, rather than being detectable in the stratum corneum.

Unexpectedly, we observed significantly lower levels of IL-8, CCL17, and CXCL10 in the skin of dialysis patients compared to healthy controls, regardless of the presence of pruritus. This finding is particularly striking given that previous studies examining the serum levels of these cytokines in CKD patients have consistently shown elevated concentrations [57]. In fact, one study even found that elevated serum levels of CCL17 are associated with pruritus in peritoneal dialysis [58]. A possible explanation for our findings, in this cohort with predominantly hemodialysis patients, is that CCL17 may be removed during hemodialysis, as it has a small molecular weight and could potentially be filtered out [59, 60]. However, this explanation does not seem applicable to IL-8 and CXCL10, where the first is typically found to be elevated in the serum of dialysis patients and the latter is associated with progression of various renal diseases [61, 62]. Another hypothesis is that immune dysregulation within the skin could significantly reduce the production of these cytokines locally, while systemic levels remain elevated. Future studies will be necessary to determine whether this is indeed the case and to explore the potential implications of reduced skin concentrations of these two cytokines.

We believe the major strength of this study lies in its approach of examining multiple inflammatory biomarkers directly in the skin of dialysis patients. While previous studies have mainly focused on the relationship between pruritus and individual cytokines, often measured systemically in serum samples, our study utilized a unique tape-stripping method to examine the outer layer of the affected skin site in patients with and without pruritus [18–21]. This non-invasive technique not only provides direct insight into the local inflammatory response but also allows for comparisons between the affected area and the non-affected site within the same patient. Other studies that have explored skin changes primarily relied on (deeper) invasive skin biopsies, often only of the affected site, thus limiting the ability to distinguish between these potential local and systemic differences [54, 63].

This study also has a few limitations. First, we selected biomarkers strongly associated with pruritus-related skin diseases, such as psoriasis and atopic dermatitis. This approach allowed us to make meaningful comparisons with these diseases and analyze potential overlapping pathophysiological mechanisms. However, this focused selection of biomarkers may have provided only a limited view of the complex interplay within the skin of dialysis patients with CKDaP. This is further reflected in the observation that, for several of the analyzed biomarkers, the measured levels showed substantial similarity between patients with and without pruritus. Nevertheless, the current selection has offered valuable insights into this overlap and can serve as a foundation for future studies. Second, the sample size, combined with adjustments for multiple testing, increased the Type II error risk (i.e., rejecting a possible effect) [64]. To address this, we also reported statistical effect sizes to provide additional context and direction for interpreting the findings. However, as this is an exploratory study, the reported effect sizes are intended to aid interpretation but should be interpreted with caution due to the limited sample size and consequent uncertainty in the estimates. Furthermore, it would be of interest to investigate whether pruritus severity is associated with the levels of the identified biomarkers. However, the current sample size did not provide sufficient statistical power to detect potential differences across levels of pruritus severity. Future studies with larger sample sizes could provide further insight into potential severity-dependent relationships. Finally, to place the differences in skin biomarker profiles between dialysis patients with and without pruritus into a broader context, we utilized samples from healthy controls obtained from a separate study. This approach carries a potential risk of introducing bias due to so-called batch differences. Previous studies on inter-batch reliability using the MSD technique have demonstrated that these differences can depend on the specific cytokine or chemokine being analyzed [65, 66]. However, based on these studies, the inter-batch correlations of the biomarkers used in our analysis appear to be generally high, suggesting that the likelihood of bias introduced by batch differences is minimal.

In conclusion, there is evidence of altered biomarker composition in the skin of dialysis patients with pruritus, highlighting inflammation as a key contributor to disease pathophysiology. Further identifying these biomarkers could inform targeted therapeutic strategies, such as anti-interleukin therapies (e.g., anti-IL-6 or anti-IL-31 antibodies), which may more precisely address the underlying mechanisms and potentially provide relief for this difficult-to-treat condition. However, before these treatments are applied, future studies with larger cohorts should focus on the relationship between pruritus severity and both local and systemic biomarkers, as well as the potential link between elevated skin biomarkers and dysregulation of the opioid receptor system.

## Supplementary Information

Below is the link to the electronic supplementary material.

Supplementary file1 (PDF 729 KB)

## Data Availability

All data are available in the manuscript or in the Supplementary Material. Raw data sets and analysis code are available upon reasonable request to the corresponding author.
