# Supplementary material for: Exploring novel skin biomarkers related to pruritus in dialysis patients
Source: Int Urol Nephrol. 2026 May 9;58(11):4965–77. doi: 10.1007/s11255-026-05169-2 (PMC13694531; doi:10.1007/s11255-026-05169-2)
Supplement: Supplementary file 1 [file 11255_2026_5169_MOESM1_ESM.pdf]

Supplementary material

Table of Contents

Table S1 2
Table S2 4
Figure S1 5
Table S3 6
Table S4 7
Table S5 9
Figure S2 10
Table S6 11
Table S7 12

Table S1 STROBE statement

STROBE Statement—Checklist of items that should be included in reports of ***cross-sectional studies***

	Item No	Recommendation	
**Title and abstract**	1	(*a*) Indicate the study's design with a commonly used term in the title or the abstract	✓
		(*b*) Provide in the abstract an informative and balanced summary of what was done and what was found	✓
**Introduction**			
Background/rationale	2	Explain the scientific background and rationale for the investigation being reported	✓
Objectives	3	State specific objectives, including any prespecified hypotheses	✓
**Methods**			
Study design	4	Present key elements of study design early in the paper	✓
Setting	5	Describe the setting, locations, and relevant dates, including periods of recruitment, exposure, follow-up, and data collection	✓
Participants	6	(*a*) Give the eligibility criteria, and the sources and methods of selection of participants	✓
Variables	7	Clearly define all outcomes, exposures, predictors, potential confounders, and effect modifiers. Give diagnostic criteria, if applicable	✓
Data sources/ measurement	8*	For each variable of interest, give sources of data and details of methods of assessment (measurement). Describe comparability of assessment methods if there is more than one group	✓
Bias	9	Describe any efforts to address potential sources of bias	✓
Study size	10	Explain how the study size was arrived at	*NA (exploratory)*
Quantitative variables	11	Explain how quantitative variables were handled in the analyses. If applicable, describe which groupings were chosen and why	✓
Statistical methods	12	(*a*) Describe all statistical methods, including those used to control for confounding	✓
		(*b*) Describe any methods used to examine subgroups and interactions	✓
		(*c*) Explain how missing data were addressed	✓
		(*d*) If applicable, describe analytical methods taking account of sampling strategy	✓
		(*e*) Describe any sensitivity analyses	*NA*
**Results**			
Participants	13*	(a) Report numbers of individuals at each stage of study—eg numbers potentially eligible, examined for eligibility, confirmed eligible, included in the study, completing follow-up, and analysed	✓
		(b) Give reasons for non-participation at each stage	✓
		(c) Consider use of a flow diagram	*NA*

Descriptive data	14*	(a) Give characteristics of study participants (eg demographic, clinical, social) and information on exposures and potential confounders	✓
		(b) Indicate number of participants with missing data for each variable of interest	✓
Outcome data	15*	Report numbers of outcome events or summary measures	✓
Main results	16	(*a*) Give unadjusted estimates and, if applicable, confounder-adjusted estimates and their precision (eg, 95% confidence interval). Make clear which confounders were adjusted for and why they were included	✓
		(*b*) Report category boundaries when continuous variables were categorized	✓
		(*c*) If relevant, consider translating estimates of relative risk into absolute risk for a meaningful time period	✓
Other analyses	17	Report other analyses done—eg analyses of subgroups and interactions, and sensitivity analyses	✓
**Discussion**			
Key results	18	Summarise key results with reference to study objectives	✓
Limitations	19	Discuss limitations of the study, taking into account sources of potential bias or imprecision. Discuss both direction and magnitude of any potential bias	✓
Interpretation	20	Give a cautious overall interpretation of results considering objectives, limitations, multiplicity of analyses, results from similar studies, and other relevant evidence	✓
Generalisability	21	Discuss the generalisability (external validity) of the study results	✓
**Other information**			
Funding	22	Give the source of funding and the role of the funders for the present study and, if applicable, for the original study on which the present article is based	✓

*Give information separately for exposed and unexposed groups.
NA. Not applicable

Note: An Explanation and Elaboration article discusses each checklist item and gives methodological background and published examples of transparent reporting. The STROBE checklist is best used in conjunction with this article (freely available on the Web sites of PLoS Medicine at http://www.plosmedicine.org/, Annals of Internal Medicine at http://www.annals.org/, and Epidemiology at http://www.epidem.com/). Information on the STROBE Initiative is available at www.strobe-statement.org.

Table S2 Selected biomarkers with their role within the skin and association with pruritic skin diseases

Biomarker	Full name	Type	Role within the skin	Skin disease associations
IL-1α	Interleukin-1 alpha	Pro-inflammatory cytokine	Expressed in keratinocytes and involved in inflammatory responses, maintaining, skin homeostasis and wound healing	Dysregulation is associated with psoriasis, atopic dermatitis and contact dermatitis
IL-1RA	Interleukin-1 receptor antagonist	Anti-inflammatory cytokine binding to IL-1 receptors	Regulating inflammatory responses and maintaining skin homeostasis	Altered expression is seen in psoriasis and atopic dermatitis
IL-8	Interleukin-8	Pro-inflammatory chemokine	Attracting neutrophils and aiding in pathogen clearance	Elevated levels are observed in psoriasis and atopic dermatitis
IL-18	Interleukin-18	Pro-inflammatory cytokine	Inducing the production of IFN-y to enhance innate and adaptive immune responses	Increased with several inflammatory skin diseases
IL-21	Interleukin-21	Immune modulating cytokine	Modulating immune response in the skin by influencing the function of T- and B-cells	Elevated IL-21 levels are seen in active psoriatic skin lesions
IL-31	Interleukin-31	Pro-inflammatory cytokine	Promoting inflammatory responses and acting on sensory neurons in the skin (inducing pruritus)	High levels are associated with several pruritic skin conditions
IFN-β	Interferon-beta	Pro-inflammatory (antiviral) cytokine	Inducing the production of other interferons in keratinocytes, particular for antiviral responses	High levels of IFN-β due to IFN-β therapy are associated with (pruritic) skin reactions
CCL17	CC chemokine ligand 17	Pro-inflammatory chemokine	Produced by dendritic cells and keratinocytes to attract T cells in context of immune response	Associated with various pruritic skin conditions, like: T-cell lymphoma, scabies and atopic dermatitis
CXCL10	CXC motif chemokine ligand 10	Pro-inflammatory chemokine	Attracting Th1 cells to sites of inflammation to enhance immune surveillance and future response	Increased expression is seen in psoriasis and atopic dermatitis

Th1, T-helper 1 cells

Figure S1 Descriptive statistics on skin biomarkers among dialysis patients with and without pruritus and healthy controls

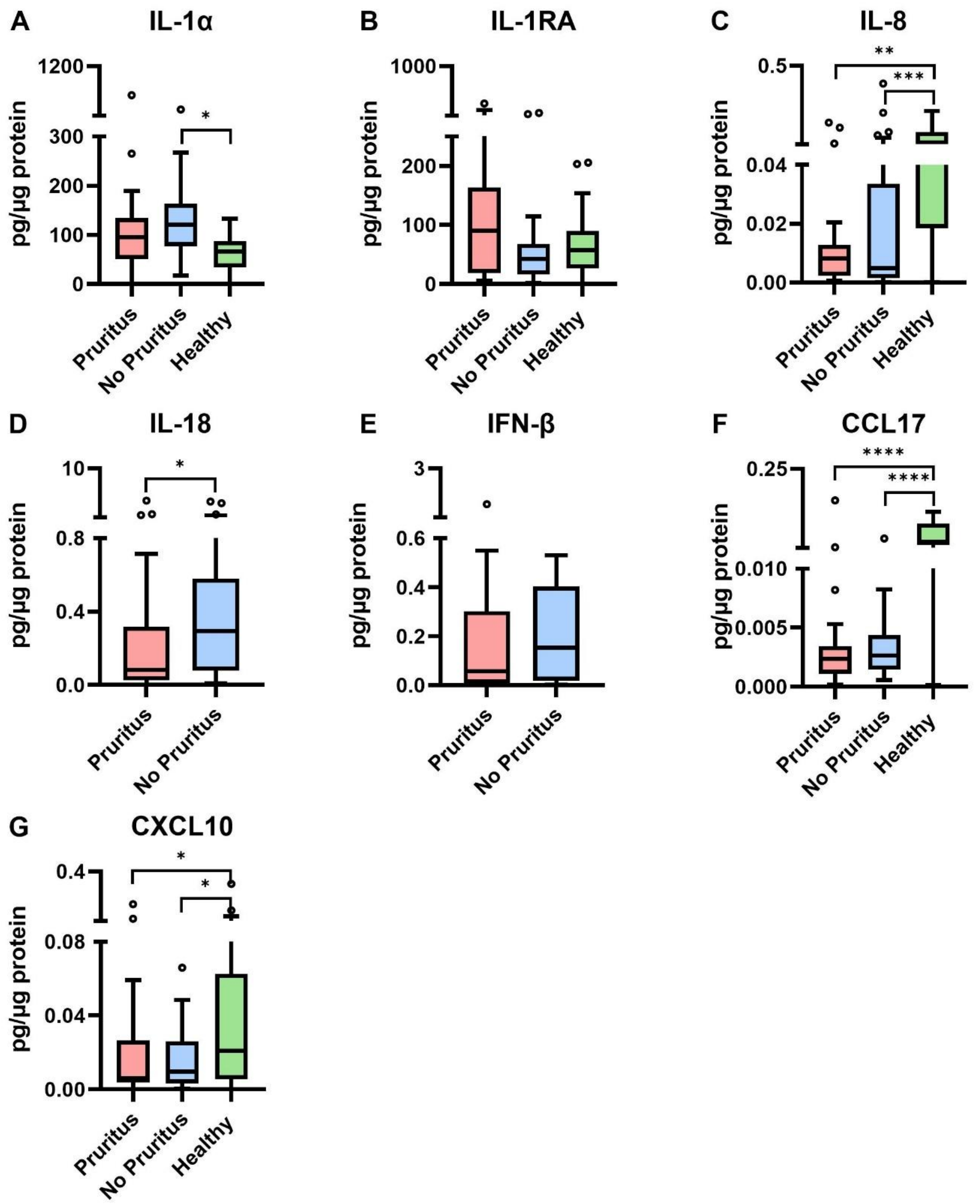

Figure S1. Distribution and differences of skin biomarkers among dialysis patients with and without pruritus and healthy controls. The skin biomarker profile for dialysis patients (n = 67) presented above included: IL-1α, n = 67 **(A)**; IL-1RA, n = 67 **(B)**; IL-8, n = 56 **(C)**; IL-18, n = 67 **(D)**; IFN-β, n = 26 **(E)**; CCL17, n = 61 **(F)**; CXCL10, n = 39 **(G)**. Patient experiencing pruritus are represented in red and those without pruritus in blue. The skin biomarker profile for healthy controls (n = 24), represented in green, included: IL-1α, n = 24 **(A)**; IL-1RA, n = 24 **(B)**; IL-8, n = 24 **(D)**; CCL17, n = 24 **(E)**; CXCL10, n = 24 **(G)**. Data are shown as medians with interquartile ranges for all values above the limit of detection. Differences between patients with pruritus, those without pruritus, and healthy controls were analyzed using an adjusted linear regression model, except for IFN-β and CXCL10 due to high percentages below limit of detection (LOD). P-values were adjusted for multiple comparisons using the Benjamini-Hochberg procedure. Statistical significance: ∗, p<0.05; ∗ ∗ , p<0.01; **p<0.001; ***p<0.0001.

IL-1α, Interleukin 1-alpha; IL-1RA, interleukin-1 receptor antagonist; IL-8, interleukin 8; IL-18, interleukin 18; IFN-β, interferon beta; CCL17, CC motif chemokine ligand 17; CXCL10, CXC motif chemokine ligand 10.

Table S3 Descriptive statistics on skin biomarkers among dialysis patients with and without pruritus and healthy controls

Skin biomarker	Dialysis patients with pruritus		Dialysis patients without pruritus		Healthy controls	
	Median	Interquartile range	Median	Interquartile range	Median	Interquartile range
IL-1α	94.919	[50.740 - 134.237]	120.397	[76.787 - 163.150]	66.469	[33.960 - 86.777]
IL-1RA	90.082	[18.739 - 163.599]	42.555	[16.494 - 68.300]	57.205	[26.702 - 90.227]
IL-8	0.008	[0.002 - 0.13]	0.005	[0.001 - 0.034]	0.050	[0.018 - 0.111]
IL-18	0.083	[0.026 - 0.318]	0.293	[0.079 - 0.580]	*NA*	*NA*
IFN-β	0.056	[0.007 - 0.302]	0.154	[0.019 - 0.403]	*NA*	*NA*
CCL17	0.002	[0.001 - 0.003]	0.003	[0.001 - 0.004]	0.038	[0.028 - 0.091]
CXCL10	0.006	[0.004 - 0.026]	0.010	[0.003 - 0.026]	0.021	[0.005 - 0.063]

Data are shown as medians with interquartile ranges for all values above the limit of detection
NA, not available; IL-1α, Interleukin 1-alpha; IL-1RA, interleukin-1 receptor antagonist; IL-8, interleukin 8; IL-18, interleukin 18; IFN-β, interferon beta; CCL17, CC motif chemokine ligand 17; CXCL10, CXC motif chemokine ligand 10

Table S4 Analyses outcomes of skin biomarkers among dialysis patients with and without pruritus and healthy controls

Analysis	Biomarker	Unadjusted model			Adjusted model			Transformed statistical effect		Adjusted *p*-value
		β-coefficient	95%CI interval	*p*-value	β-coefficient	95%CI interval	*p*-value	Effect	95% CI interval	
Dialysis patients with pruritus compared to patients without pruritus										
	IL-1α	-0.268	(-0.581; 0.045)	0.092	-0.314	(-0.637; 0.009)	0.057	0.731α	(0.529; 1.009)	0.158
	IL-1RA	0.621	(0.047; 1.194)	0.034	0.524	(-0.048; 1.095)	0.072	1.688[a]	(0.954; 2.988)	0.158
	IL-8	-0.119	(-1.261; 1.023)	0.836	-0.026	(-1.216; 1.163)	0.965	0.974[a]	(0.297; 3.199)	0.965
	IL-18	-1.081	(-1.891; -0.271)	0.0098	-1.191	(-1.931; -0.450)	0.0021	0.304[a]	(0.145; 0.637)	0.015
	IFN-β	1.121	(0.030; 2.283)	0.049	0.981	(-0.189; 2.216)	0.106	2.667[b]	(0.827; 9.168)	0.877
	CCL17	-0.575	(-1.195; 0.045)	0.069	-0.517	(-1.161; 0.126)	0.113	0.596[a]	(0.313; 1.135)	0.158
	CXCL10	0.08	(-0.957; 1.122)	0.879	0.174	(-0.901; 1.266)	0.751	1.19[b]	(0.406; 3.547)	0.158
Dialysis patients with pruritus compared to healthy controls										
	IL-1α	0.529	(0.055; 1.003)	0.029	0.499	(-0.038; 1.036)	0.068	1.647[a]	(0.963; 2.818)	0.085
	IL-1RA	0.181	(-0.482; 0.8423)	0.588	0.396	(-0.345; 1.137)	0.291	1.486[a]	(0.708; 3.117)	0.291
	IL-8	-3.039	(-4.177; -1.901)	<0.0001	-3.135	(-4.424; -1.846)	<0.0001	0.044[a]	(0.012; 0.158)	<0.0001
	CCL17	-3.436	(-4.168; -2.703)	<0.0001	-3.437	(-4.266; -2.608)	<0.0001	0.032[a]	(0.014; 0.074)	<0.0001
	CXCL10	-3.555	(-6.507; -0.603)	0.0182	-3.612	(-6.52338; -0.700)	0.015	0.027[b]	(0.001; 0.497)	0.025
Dialysis patients without pruritus compared to healthy controls										
	IL-1α	0.797	(0.326; 1.267)	0.001	0.773	(0.207; 1.340)	0.008	2.166[a]	(1.230; 3.819)	0.014
	IL-1RA	-0.44	(-1.098; 0.218)	0.187	-0.172	(-0.954; 0.610)	0.663	0.842[a]	(0.385; 1.840)	0.663
	IL-8	-2.921	(-4.05; -1.791)	<0.0001	-3.057	(-4.417; -1.697)	<0.0001	0.047[a]	(0.012; 0.183)	<0.0001
	CCL17	-2.861	(-3.587; -2.134)	<0.0001	-2.889	(-3.763; -2.013)	<0.0001	0.056[a]	(0.023; 0.134)	<0.0001
	CXCL10	-3.632	(-6.580; -0.684)	0.0157	-3.77	(-6.726; -0.814)	0.012	0.023[b]	(0.001; 0.443)	0.016

IL-1α, IL-1RA, IL-8, IL-18 and CCL17 were log-transformed before analyzing due to skewed distribution (of residuals), and were analyzed using a linear regression model. IFN-β and CXCL10 were dichotomized in detectable and non-detectable due to a large amount of values below detection limit and were analyzed using a logistic regression model. Overall statistical effects were calculated by exponent-transformation of the β-coefficients into proportion differences (in case of linear regression) and odds ratios (in case of logistic regression). All adjusted models were adjusted for the following potential confounders: age, sex, immunosuppressant use and atopy. The Benjamini-Hochberg procedure was used to adjust for multiple testing. A p-value of <0.05

was considered statistically significant. a, proportional difference; b, odds ratio. CI, confidence interval; IL-1α, interleukin 1-alpha; IL-1RA, interleukin-1 receptor antagonist; IL-8, interleukin 8; IL-18, interleukin 18; IFN-β, interferon beta; CCL17, CC motif chemokine ligand 17; CXCL10, CXC motif chemokine

Table S5 Proportion of values below limit of detection

Biomarker	Lower LOD (pg/mL)	Percentage below LOD			
		Dialysis patients			Healthy controls
		Total	Affected/torso site	Non-affected (control) site	Back site
IL-1α	50.58503243	0.0	0.0	0.0	0.0
IL-1RA	25.19970622	0.0	0.0	0.0	0.0
IL-8	0.001375125	14.9	16.4	13.4	0.0
IL-18	0.013860142	0.0	0.0	0.0	0.0
IL-21	*NA*	100.0	100.0	100.0	0.0
IL-31	0.15626012	95.5	95.5	95.5	0.0
IFN-β	0.004255987	62.0	61.2	62.7	0.0
CCL17	0.000298222	16.5	9.0	23.9	0.0
CXCL10	0.001224096	43.3	41.8	44.8	0.0

LOD, limit of detection; NA, not available, IL-1α, Interleukin 1-alpha; IL-1RA, interleukin-1 receptor antagonist; IL-8, interleukin 8; IL-18, interleukin 18; IL-21, interleukin 21; IL-31, interleukin 31 IFN-β, interferon beta; CCL17, CC motif chemokine ligand 17; CXCL10, CXC motif chemokine ligand 10

Figure S2 Distribution and differences of skin biomarkers of the affected and non-affected site within dialysis patients with pruritus

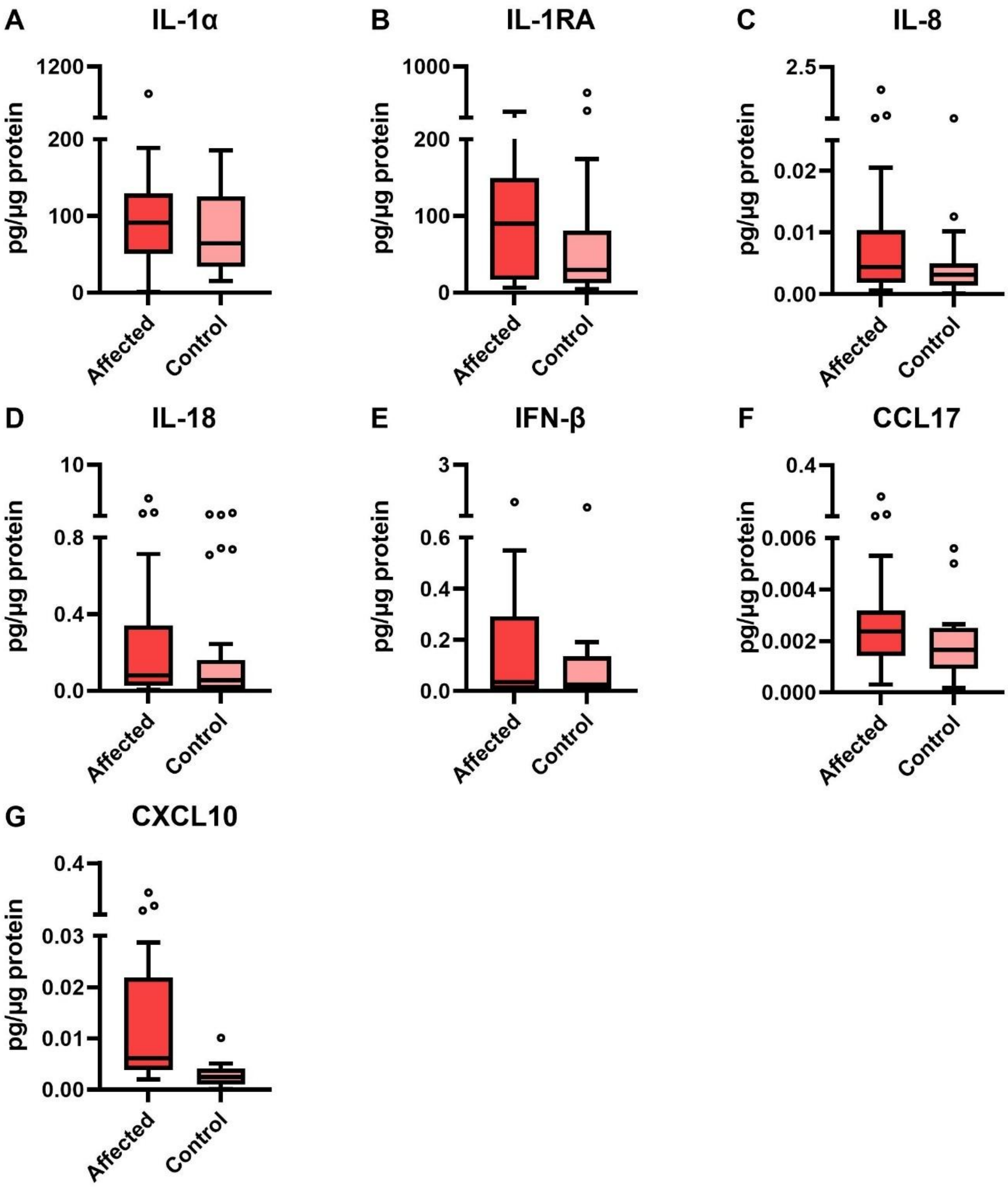

Figure S2. Distribution and differences of skin biomarkers of the affected and non-affected site within dialysis patients with pruritus. The skin biomarker profile in both sites in dialysis patients with pruritus (n = 34), presented above, included: IL-1α, n = 68 **(A)**; IL-1RA, n = 68 **(B)**; IL-8, n = 62 **(C)**; IL-18, n = 68 **(D)**; IFN-β, n = 33 **(E)**; CCL17, n = 52 **(F)**; CXCL10, n = 37 **(G)**. The affected site is represented in red and the non-affected site in pink. Data are shown as medians with interquartile ranges for all values above the limit of detection. Differences in biomarkers between the sites were analyzed using an adjusted linear mixed model, except for IFN-β and CXCL10 due to high percentages below limit of detection (LOD). Models were adjusted for sex, age, use of immunosuppressants, the presence of atopy, and a random intercept for participants. Four patients were left out of analysis due to experiencing pruritus on both sites. p-values were adjusted for multiple comparisons using the Benjamini-Hochberg procedure. Statistical significance: ∗, p<0.05; ∗ ∗ , p<0.01; **p<0.001; ***p<0.0001.

IL-1α, Interleukin 1-alpha; IL-1RA, interleukin-1 receptor antagonist; IL-8, interleukin 8; IL-18, interleukin 18; IFN-β, interferon beta; CCL17, CC motif chemokine ligand 17; CXCL10, CXC motif chemokine ligand 10.

Table S6 Descriptive statistics on skin biomarkers of the affected and non-affected site within dialysis patients with and without pruritus

Skin biomarker	Dialysis patients with pruritus				Dialysis patients without pruritus			
	Affected site		Control site (non-affected)		Torso site		Control site	
	Median	Interquartile range	Median	Interquartile range	Median	Interquartile range	Median	Interquartile range
IL-1α	94.919	[50.740 - 134.237]	67.514	[39.805 - 125.622]	120.397	[76.787 - 163.150]	73.827	[54.466 - 114.205]
IL-1RA	90.082	[18.739 - 163.599]	38.975	[12.867 - 115.198]	42.555	[16.494 - 68.300]	25.231	[12.937 - 76.598]
IL-8	0.008	[0.002 - 0.13]	0.003	[0.002 - 0.008]	0.005	[0.001 - 0.034]	0.005	[0.002 - 0.015]
IL-18	0.083	[0.026 - 0.318]	0.054	[0.011 - 0.360]	0.293	[0.079 - 0.580]	0.198	[0.375 - 0.501]
IFN-β	0.056	[0.007 - 0.302]	0.048	[0.009 - 0.175]	0.154	[0.019 - 0.403]	0.044	[0.007 - 0.246]
CCL17	0.002	[0.001 - 0.003]	0.0016	[0.0009 - 0.0025]	0.003	[0.001 - 0.004]	0.0013	[0.0008 - 0.0034]
CXCL10	0.006	[0.004 - 0.026]	0.003	[0.001 - 0.005]	0.010	[0.003 - 0.026]	0.009	[0.006 - 0.018]

Data are shown as medians with interquartile ranges for all values above the limit of detection

IL-1α, Interleukin 1-alpha; IL-1RA, interleukin-1 receptor antagonist; IL-8, interleukin 8; IL-18, interleukin 18; IFN-β, interferon beta; CCL17, CC motif chemokine ligand 17; CXCL10, CXC motif chemokine ligand 10

Table S7 Analyses outcomes of skin biomarkers of the affected and non-affected site within dialysis patients with pruritus

Analysis	Biomarker	Unadjusted model			Adjusted model			Statistic effect		Adjusted *p*-value
		β-coefficient	95%CI interval	*p*-value	β-coefficient	95%CI interval	*p*-value	Effect	95% CI interval	
Affected site compared to non-affected site in dialysis patients with pruritus										
	IL-1α	0.257	(0.009; 0.505)	0.047	0.236	(-0.012; 0.484)	0.076	1.266[a]	(0.989; 1.623)	0.177
	IL-1RA	0.601	(0.154; 1.049)	0.012	0.607	(0.161; 1.052)	0.0137	1.835[a]	(1.175; 2.865)	0.062
	IL-8	-0.137	(-0.857; 0.582)	0.703	-0.235	(-0.971; 0.502)	0.541	0.791[a]	(0.379; 1.652)	0.541
	IL-18	0.401	(-0.426; 1.228)	0.336	0.348	(-0.490; 1.186)	0.427	1.416[a]	(0.613; 3.275)	0.498
	IFN-β	0.751	(-1.04; 2.544)	0.411	0.991	(-1.236; 3.219)	0.383	2.694[b]	(0.290; 25.006)	0.498
	CCL17	0.824	(0.190; 1.459)	0.0145	0.834	(0.196; 1.472)	0.0176	2.302[a]	(1.216; 4.356)	0.062
	CXCL10	0.505	(-0.703; 1.713)	0.413	0.51	(-0.730: 1.750)	0.424	1.666[b]	(0.481; 6.261)	0.498

IL-1α, IL-1RA, IL-8, IL-18 and CCL17 were log-transformed before analyzing due to skewed distribution (of residuals), and were analyzed using a linear mixed-effects model. IFN-β and CXCL10 were dichotomized in detectable and non-detectable due to a large amount of values below detection limit and were analyzed using a logistic mixed-effects model. Overall statistical effects were calculated by exponent-transformation of the β-coefficients into proportion differences (in case of linear mixed-effects models) and odds ratios (in case of logistic mixed-effects models). All adjusted models were adjusted for the following potential confounders: age, sex, immunosuppressant use and atopy with a random intercept on patient level. The Benjamini-Hochberg procedure was used to adjust for multiple testing. A p-value of <0.05 was considered statistically significant. a, proportional difference; b, odds ratio. CI, confidence interval; IL-1α, Interleukin 1-alpha; IL-1RA, interleukin-1 receptor antagonist; IL-8, interleukin 8; IL-18, interleukin 18; IFN-β, interferon beta; CCL17, CC motif chemokine ligand 17; CXCL10, CXC motif chemokine ligand
